# Pediatric Febrile Urinary Tract Infection Caused by ESBL Producing *Enterobacteriaceae* Species

**DOI:** 10.1155/2020/6679029

**Published:** 2020-12-02

**Authors:** Asnakech Agegnehu, Mesfin Worku, Demiss Nigussie, Birhanu Lulu, Birkneh Tilahun Tadesse

**Affiliations:** ^1^Microbiology Laboratory, Hawassa University Comprehensive Specialized Hospital, Ethiopia; ^2^School of Medical Laboratory Science, College of Medicine and Health Sciences, Hawassa University, Ethiopia; ^3^Department of Medical Laboratory Science, College of Medicine, Medicine and Health Sciences institute, Debrebirhan University, Ethiopia; ^4^Department of Pediatrics, School of Medicine, College of Medicine and Health Sciences, Hawassa University, Ethiopia

## Abstract

**Background:**

Over the past decade, drug resistance pattern has worsened for many of the uropathogens due to overuse of antibiotics for empiric treatment. The burden of extended spectrum beta-lactamase (ESBL) producing *Enterobacteriaceae* associated urinary tract infections (UTI) has become increasingly more common, limiting treatment options among children presenting with febrile UTI. We investigated the burden and correlates of ESBL producing *Enterobacteriaceae* associated UTI among children and antibacterial resistance pattern.

**Methods:**

284 midstream urine specimens were collected using standard aseptic techniques from 284 children who were diagnosed with suspected UTI. Urine culture and bacteria isolation were performed following standard bacteriological techniques. The Kirby-Bauer disk diffusion technique and the double-disc synergy test were used to investigate antibiotic susceptibility and presence of ESBL production.

**Results:**

UTI was confirmed using a positive urine culture for a relevant pathogen in 96/284 (33.8%) of the cases. *Enterobacteriaceae* accounted for 75% (72/96) of etiologies of UTI in children. The most frequent *Enterobacteriaceae* spp. were *E. coli*, 44.4% (32/72) and *K. pneumonia*, 27.8% (20/72). The overall multidrug resistance rate was 86.1% (62/72). ESBL-producers accounted for 41.7% (30/72) of the isolated *Enterobacteriaceae*. ESBL producing *K. pneumonia* and *E. coli* isolates accounted for 70% (14/20) and 37.5% (12/32), respectively. History of UTI in the past 1 year (adjusted odds ratio (AoR) = 0.08, 95%CI (0.01 − 0.57)) and medium family wealth index (AoR = 0.03, 95%CI (0.00 − 0.27)) protected from infection with ESBL-producing *Enterobacteriaceae. Conclusion*. ESBL production was more common in *K. pneumonia* and appeared to be a major factor contributing drug resistance UTI in children. The findings call for the need to incorporate ESBL testing in the routine clinical practice. The resistance level to commonly prescribed first-line antibiotics observed within *Enterobacteriaceae* was alarming calling for strengthened antimicrobial stewardship.

## 1. Introduction

Urinary tract infection (UTI), which is commonly caused by *Enterobacteriaceae* like *Escherichia coli* (*E. coli*) and *Klebsiella species* and other Gram-negative bacteria, is one of the commonest causes of febrile illnesses in children [1, 2]. Extended spectrum *β*-lactamase (ESBL) producing *Enterobacteriaceae* causing UTI have been associated with prescription of broad-spectrum antibiotics, which further exacerbate the challenge of antimicrobial resistance (AMR) [3].

In several settings, the occurrence of ESBL-producing bacterial infections like *E. coli* and *Klebsiella species* were described as a pandemic [1, 4, 5]. ESBLs are plasmid-mediated enzymes, which confer resistance to Gram-negative bacteria [6], through three mechanisms—first, *β*-lactam cannot reach penicillin-binding proteins (PBP); second, decreased affinity to PBPs; and third, *β*-lactamases destroy the drug [7]. *β*-lactamase enzymes destroy inactivate *β*-lactam containing antibiotics, and ESBLs contain serine. These enzymes can hydrolyze penicillin, cephalosporin, aztreonam, and monobactam antibiotics making them ineffective. Cephamycin groups including cefoxitin and cefotetan and carbapenems including imipenem, meropenem, and ertapenem are not hydrolyzed by ESBLs and are hence considered as the drugs of choice for treating ESBL-producing *Enterobacteriaceae* [8]. However, these drugs are not readily available; and, in settings where diagnostics for ESBL producing *Enterobacteriaceae* are limited, targeting treatment becomes a challenge.

Circulation of ESBL-producing *Enterobacteriaceae* in the community and healthcare settings is a significant global challenge as this could be associated with increasing trends of AMR, which is even more significant in the sub-Saharan African region [9]. Furthermore, high levels of AMR related to ESBL-producing *Enterobacteriaceae* complicate individual patient care increasing the mortality and morbidity associated with common infectious diseases in children like UTI [10]. Of note, the alarming AMR trend is partly due to the irrational use of antibiotics in resource-limited settings which further leads to more selective pressure drug resistance [7, 11].

There is limited data on the extent of infections among children caused by ESBL-producing *Enterobacteriaceae*. We investigated the prevalence and correlates of pediatric UTI caused by ESBL-producing *Enterobacteriaceae* at a tertiary hospital. The findings will have a critical importance to guide empiric use of antibiotics in the pediatric population in the setting. It will also shed light on the importance of routine testing for ESBL-production in pediatric urine samples.

## 2. Methods and Materials

### 2.1. Study Setting and Participants

A cross-sectional study was conducted from February 1, 2018, to July 30, 2018, among children presenting to the outpatient department of Hawassa Comprehensive Specialized Hospital, which is a tertiary referral facility in southern Ethiopia.

Included were children below the age of 15 years with clinically suspected UTI defined as those with at least one of the signs and symptoms of urinary tract infection including frequency, urgency, dysuria, abdominal pain, back pain, and fever (>38.0°C). Participants who received antibiotics within two weeks before presentation to the hospital were excluded.

### 2.2. Sample Size

Sample size was calculated using single-population proportion formulae considering the prevalence of extended spectrum *β*-lactamase (ESBL) producing *Enterobacteriaceae* of 78.6% [12]. Using survey methods, 10% nonresponse rate and 95% confidence interval, a final sample size of 284 subjects with suspected UTI was estimated.

### 2.3. Data Collection

#### 2.3.1. Sociodemographic and Clinical Data

Sociodemographics including age (years), sex, residence, parental education and occupation, living standard, and clinical history of participants such as hospital admission and history of UTI within the past 12 months were recorded.

#### 2.3.2. Laboratory Data Collection

A total of 284 early morning midstream (MSU) and catheterized urine samples were collected using properly labelled sterile, clean, transparent, screw-capped, wide-mouth plastic cups. Samples were transported to the microbiology laboratory within two hours of collection. Standard wire loop of 1 *μ*l diameter was used to inoculate approximately 0.001 mL urine on 5% sheep Blood agar (OXOID Ltd. England) and MacConkey agar (OXOID Ltd., Basingstoke, United Kingdom) plate which were incubated aerobically at 37°C for 24 hrs.

Significant bacterial growth was determined on Blood agar plate when a single midstream or catheterized urine culture yields >10^5^ CFU/mL [13]. Macroscopic examination for hemolysis, changes in physical appearance on differential media, and colony characteristics were recorded to help in identification. Furthermore, Gram reaction, morphology, and colony arrangement were recorded. Biochemical tests including Indole production, sugar fermentation, H_2_S and gas production, citrate utilization, motility test, mannitol test, and urease and oxidase test were also done to further identify *Enterobacteriaceae* isolates. If not processed immediately, we kept the isolated bacteria at 2–8°C in a nutrient broth for not more than 24 hrs until the antimicrobial sensitivity test was done.

### 2.4. Detection and Confirmation of ESBL Producing Enterobacteriaceae

Standard disc diffusion method was used to assess ESBL production among isolated *Enterobacteriaceae* which was then confirmed by the double-disc synergy test (DDST) using third-generation cephalosporins and modified double-disc synergy test using cefepime along with the third-generation cephalosporins, following standard microbiological procedures and the Clinical and Laboratory Standards Institute (CLSI) guidelines [14].

### 2.5. Procedures for Double-Disc Synergy Test (DDST)

Antibiotic discs of 20/10 *μ*g of amoxicillin/clavulanic acid centrally and 30 *μ*g each of cefotaxime and ceftazidime were placed center to center at 15 mm apart, followed by incubation at 37°C for 18-24 hours. ESBL production was considered when inhibition zones of cefotaxime and ceftazidime extending towards clavulanic acid disc. Modified double-disc synergy test using Cefepime antibiotic disc was used to further detect AMPC lactamase coproducing false-negative isolates.

### 2.6. Procedures for Modified Double-Disc Synergy Test (MDDST)

Similar concentrations of amoxicillin-clavulanate antibiotic disc with cephalosporins namely ceftriaxone, cefotaxime, cefepime, and ceftazidime are used in MDDST. Any distortion or increase in the zone of inhibition of the cephalosporin discs towards the amoxicillin-clavulanate disc was considered as confirmatory for ESBLs production.

### 2.7. Antimicrobial Susceptibility Testing

Kirby-Bauer disk diffusion method was used to assess antimicrobial susceptibility as recommended by the CLSI guidelines [13, 14]. Ampicillin (AMP: 10 *μ*g), ciprofloxacin (CEP: 30 *μ*g), nitrofurantoin (NIF: 300 *μ*g, BD), norfloxacin (NOR: 10 *μ*g), amoxicillin-clavulanic acid (AMC: 20/10 *μ*g), gentamicin (G: 10 *μ*g), trimethoprim-sulfamethoxazole (STX: 1.25/23.75 *μ*g) cefotaxime (CTX: 30 *μ*g), cefoxitin (FOR: 30 *μ*g), ceftriaxone (CTR: 30 *μ*g), tetracycline (TE: 30 *μ*g), and meropenem (MEM: 10 *μ*g). Zone of inhibition was measured after overnight incubation at 37°C. Nonsusceptibility to three or more classes of antibiotics defined multidrug resistance.

### 2.8. Data Analysis

Descriptive data were presented as frequency (percentage), mean (standard deviation), median (range), and using tables and figures. Predictors of infection with ESBL producing *Enterobacteriaceae* were assessed using bivariate logistic regression. Multivariate logistic regression models were run using all variables with a **P** value < 0.25 in univariate analysis. Covariates with a **P** value of <0.05 in the multivariate models were considered as independent predictors of infection with ESBL producing bacteria. Anthropometric **Z**-scores were generated using WHO Anthro plus, while family wealth status was assessed using principal component analysis (PCA).

### 2.9. Ethical Considerations

The study received ethical approval from the institutional review board (IRB) of Hawassa University (IRB Number: IRB/156/10). Written informed assent was obtained from study participants or caretakers of children. Patient privacy was protected by deidentification of records. Names of patients were coded. All data obtained during the study were kept confidential and were used solely for the purpose of the study. Positive laboratory result from the study participant was communicated to their physicians for appropriate treatment or management.

## 3. Results

### 3.1. Demographic and Clinical Characteristics

A total of 284 children ≤14 years of age were included in the study. Of the total study participants, 52.46% (149/284) were male, 61.3% (174/284) were urban residents, 52.8% (150/284) were treated as inpatient, 20.1% (57/284) had malnutrition, and 9.9% (28/284) underwent surgical procedures in the past 6 months. Subjects with a history of hospital admission and UTI within the past 12 months accounted for 33.8% (96/284) and 14.1% (40/284), respectively (Table 1).

### 3.2. Frequency of Enterobacteriaceae Isolates

From 284 urine specimens, growth was detected in 90 specimens, and a total of 96 (33.8%) bacterial species were identified. Among these, 75% (*n* = 72/96) were *Enterobacteriaceae* with *E. coli* (44.4%, *n* = 32/72), *K. pneumonia* (27.8%, *n* = 20/72), *Klebsiella oxytoca* (8.33%, *n* = 6/72), *Providencia* spp. (5.6%, *n* = 4/72), *Citrobacter diversus* (4.16%, *n* = 3/72), *Enterobacter cloacae* (2.8%, *n* = 2/72), *Proteus mirabilis* (2.8%, *n* = 2/72), and *Klebsiella ozaenae* (4.16%, *n* = 3/72) being most common isolates. The remaining 25% (24/96) from the non-*Enterobacteriaceae* group were *Pseudomonas* spp. (3.12%, *n* = 3/96), *Enterococcus* spp. (11.5%, *n* = 11/96), *S. aureus* (5.21%, *n* = 5/96), *S. saprophyticus* (3.12%, *n* = 3/96), and yeast cell (2.1%, *n* = 2/96) (Table 2).

**Figure 1:**
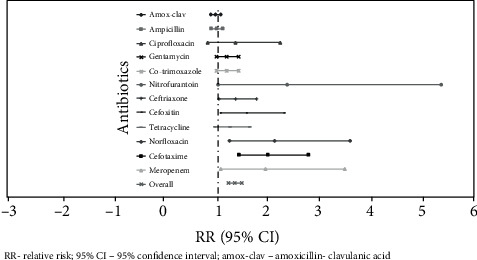
Relative risk of antibiotic resistance in ESBL producers as compared to nonproducers. RR: relative risk; 95% CI: 95% confidence interval; Amox-Clav: amoxicillin-clavulanic acid.

*Enterobacteriaceae* were more commonly isolated among female subjects (55.6%, *n* = 40/72) than male subjects (41.7%, *n* = 30/72) (*P* value = 0.07). Furthermore, *Enterobacteriaceae* were isolated from study subjects who attended inpatient department (59.7%, 43/72), urban (58.3%, 42/72), and aged less than four years (54.2%, 39/72). *Klebsiella* species, 25% (18/72), and *E. coli*, 16.7% (12/72), were the most frequently isolated *Enterobacteriaceae* (Table 2 and Figure 1).

### 3.3. Prevalence and Predictors of ESBL Producing Enterobacteriaceae

Potentially ESBL producing *Enterobacteriaceae* accounted for 58.3% (42/72) of the total isolates, of which 71.4% (30/42) were confirmed as ESBL producers.

We then performed bivariate analysis using paternal occupation, maternal education, paternal education, age, place of residence, patient type, history of UTI within the past 12 months, and family wealth index against ESBL infection. Next, we pooled the variables with a *P* value < 0.025 to identify independent predictors of infection with ESBL producing *Enterobacteriaceae*. Children with a history of UTI within the past 12 months were less likely to be infected with ESBL producing *Enterobacteriaceae* (AOR = 0.076 with 95% CI (0.010-0.569). Family wealth index of medium was associated with lower risk of infection by ESBL-producing Enterobacteriaceae as compared to those with poor family wealth index (AOR = 0.029 with 95% CI (0.003-0.265) (Table 3).

### 3.4. Antibiotic Resistance Profile of Isolated Enterobacteriaceae

The antibiotics resistance profile of *Enterobacteriaceae* isolated in urine specimen against 12 antibiotics is presented in Tables 4–6. Majority of isolates were resistant to ampicillin (95.8%), amoxicillin/clavulanic acid (94.4%), trimethoprim-sulfamethoxazole (86.1%), and gentamycin (86.1%), while better susceptibility was observed for ciprofloxacin (47.2%), norfloxacin (45.8%), meropenem (40.3%), and nitrofurantoin (26.4%). Of the 72 *Enterobacteriaceae* isolates tested for antibiotic susceptibility testing, 62 (86.1%) were nonsusceptible to three or more drugs belonging to different antibiotics classes. Four (5.6%) of the isolates were nonsusceptible to all antibiotics tested. From ESBL-producing *Enterobacteriaceae*, 96.7% were multidrug resistant.

### 3.5. Drug Resistance of ESBL-Producing Enterobacteriaceae

ESBL-producing *Enterobacteriaceae* were resistant to amoxicillin/clavulanic acid (96.7%), ampicillin (96.7%), trimethoprim-sulfamethoxazole (96.7%), gentamycin (96.7%), cefotaxime (96.7%), ceftriaxone (90%), and tetracycline (86.7%) as compared to ESBL non-producers (Table 6). Except for amoxicillin-clavulanic acid, ampicillin, and ciprofloxacin, the odds of resistance to all other tested antibiotics was significantly higher among ESBL producing *Enterobacteriaceae* as compared to nonproducers (Figure 1).

## 4. Discussion

Our findings show that urine culture growth was observed in a third of cases presenting with clinically suspected cases of UTI. The majority of bacterial isolates identified in the culture were Gram-negative bacteria, a finding comparable to findings from another tertiary care hospital in Ethiopia [12]. The culture confirmation rate of suspected cases in the current study higher than studies elsewhere [10, 15, 16], reflecting the variability in clinical index of suspicion and the healthcare settings. Important limitations of the current study include that certain clinical features such as ICU admission and circumcision (for boys) were not assessed as potential factors for infection with ESBL producing *Enterobacteriaceae*. Molecular epidemiological characterization of ESBL producing *Enterobacteriaceae* was not possible.

The prevalence of ESBLs producers within the *Enterobacteriaceae* in the study was 41.7%, which is similar to findings in Ethiopia (38.0-51%) [17, 18], Nigeria (47.1%) [19], and India (38.2%) [20]. However, the prevalence of ESBL production in the current study is much lower than that reported from a study at a tertiary facility in Ethiopia [12], which included *Enterobacteriaceae* from specimens other than urine and reported a much smaller number of isolates. The ESBL prevalence was also lower than those reported in Burkina Faso 58%, from clinical samples [21] and Nepal 62.31% from urinary isolates [22], both of which included adults. On the other hand, the prevalence of ESBL observed in our study was higher than another study in Ethiopia (25%) [23], and studies in Morocco (25.5%) [24], Iran (28.4%) [25], and Korea (16.7%) [26]. The higher prevalence seen in our study compared to developed countries like Greece (10.4%) [27] could be attributed to differences in infection control policies and practices, duration of hospitalization, and improved nursing barriers that decrease acquisition and spread of ESBL producing strains.

Similar to previous reports, *E. coli* was the most common cause of UTI followed by *K. pneumonia* [18, 28, 29]. Even though *E. coli* had high isolation rate (44.4%) in the study, *Klebsiella* spp. was identified as the major ESBL producer 25% (18/72) followed by *E. coli* 16.7% (12/72). A similar trend was reported by studies in Ethiopia and Pakistan [17, 30], while more ESBL production in *E. coli* as compared to *K. pneumonia* in another study in Ethiopia [12].

A worrying level of multidrug resistance was reported in our study which adds to previous reports in other facilities in Ethiopia, for example, 87.4% in Northwest Ethiopia [1] and 68.3% in Addis Ababa, Ethiopia [18]. Studies elsewhere also reported similarly high isolation rates of multidrug-resistant bacteria, for instance in Chicago, USA, 76% [31], Nepal 64.04% [32], and Iran 52.7% [29]. Unsurprisingly, ESBL-producing *Enterobacteriaceae* are associated with multidrug resistance in nearly all the cases (96.7%)—a finding very close to another survey in Addis Ababa, Ethiopia, with 96.3% multidrug resistance [18]. Nitrofurantoin demonstrated a lower level of resistance among *E. coli* and *K. pneumoniae* isolates, favoring the use of this antibiotic for empiric treatment of UTI in children. The lower resistance levels could point to the lower utilization of the antibiotic [33]. Our finding together with similar previous studies underscore the contribution of ESBL producing *Enterobacteriaceae spp* in multidrug resistance outbreaks in healthcare facilities. The findings call for an urgent assessment of the national burden of ESBL-producing *Enterobacteriaceae* which cause UTI and other clinical diseases including the antimicrobial susceptibility patterns. Such efforts would have paramount importance to guide local treatment and care guidelines.

Antibiotic resistance levels of *Enterobacteriaceae* are reported to be high in several resource-limited settings. For example, in Iran, resistance to trimethoprim-sulfamethoxazole (93.6%), ciprofloxacin (40.4%), and tetracycline (84.5%) [29] was reported. Similarly, in Bangladesh, 97.8% for trimethoprim-sulfamethoxazole was reported [34]. These figures are considerably lower in high-income countries, for example, in the United States, resistance to ampicillin (55%), amoxicillin/clavulanic acid (10%), trimethoprim-sulfamethoxazole (24%), and nitrofurantoin (14%) of *Enterobacteriaceae* was reported [31]. These findings present a concerning public health problem, which requires a coordinated action to generate more data in understanding the magnitude of the problem and designing interventions that help to mitigate the issue.

## 5. Conclusion

Our research finding evidenced that ESBL-producing bacteria are prevalent among children with UTI. From the total bacteria species isolated, *Enterobacteriaceae* contributed to the majority of the isolates. Most of the *Enterobacteriaceae* were isolated from patients who attended inpatient department. ESBL-producing *Klebsiella species* were the most frequent *Enterobacteriaceae* followed by *E. coli*. Majority of *Enterobacteriaceae* had resistance to commonly prescribed antibiotics. The drug with preserved efficacy for ESBL producers and non-ESBL producers is nitrofurantoin.

## Figures and Tables

**Table 1:** Demographic and clinical characteristics of study participants among UTI suspected children (<15 years) at HUCSH from February 1 to July 30, 2018, Hawassa, Ethiopia.

Variables	Category	Number	Percentage
Age	0-4 years	159	56.0
	5-9 years	61	21.5
	10-14	64	22.5
Sex	Male	149	52.5
	Female	135	47.5
Place of residence	Urban	174	61.3
	Rural	110	38.7
Patient type	Outpatient	134	47.2
	Inpatient	150	52.8
Malnourished	Yes	57	20.1
	No	227	79.9
Hospital admission within the past 12 months	Yes	96	33.8
	No	188	66.2
Surgery within the past 6 months∗	Yes	28	9.9
	No	256	90.1
UTI within the past 12 months	Yes	40	14.1
	No	244	85.9
Paternal education	No education	78	27.5
	Primary	87	30.6
	Secondary and above	119	41.9
Maternal education	No education	109	38.4
	Primary	87	30.6
	Secondary and above	88	31.0
Paternal occupation	Employed	125	44.0
	Merchant	46	16.2
	Farmer	82	28.9
	Daily laborers	31	10.9
Maternal occupation	Housewife	173	60.9
	Employed	61	21.5
	Merchant	50	17.6

**Table 2:** Frequency of ESBL producer and non-ESBL producer *Enterobacteriaceae* isolated from children suspected of UTI at HUCSH from February 1 to July 30, 2018, Hawassa, Ethiopia.

	*K. pneumonia* No (%)	*E. coli* No (%)	*K. Oxytoca* No (%)	K. Ozaenae No (%)	*E. cloacae* No (%)	*P. mirabilis* No (%)	*C. diversus* No (%)	*Providencia* spp. No (%)	Total
ESBL-producer	14 (70)	12 (37.5)	3 (50)	1 (33.3)	0 (0.0)	0 (0.0)	0 (0.0)	0 (0.0)	30 (41.7)
Non-ESBL producer	6 (30)	20 (62.5)	3 (50)	2 (66.7)	2 (100)	2 (100)	3 (100)	4 (100)	42 (58.3)
Total	20 (100)	32 (100)	6 (100)	3 (100)	2 (100)	2 (100)	3 (100)	4 (100)	72 (100)

**Table 3:** Bivariate and multivariable analysis of independent variables against ESBL production status among UTI suspected children (<15 years) at HUCSH from February 1, 2018, to July 30, 2018, Hawassa, Ethiopia.

Variable	Category	ESBL producer	ESBL nonproducer	COR (95% CI)	AOR (95% CI)	*P* value
Age	0-4	11	28	1	1	
	5-9	7	8	0.449 (0.131-1.538)	0.506 (0.078-3.261)	0.473
	10-14	12	6	0.196 (0.059-0.654)	0.183 (0.029-1.157)	0.071
Place of residence	Urban	22	20	1	1	0.142
	Rural	8	22	3.025 (1.101-8.311)	3.900 (0.634-13-984)	
Patient type	Outpatient	16	13	1	1	0.301
	Inpatient	14	29	2.549 (0.966-6.731)	2.546 (0.433-14-958)	
UTI within the past 12 months	No	20	34	1	1	0.012
	Yes	10	8	0.471 (0.160-1.388)	0.076 (0.010-0.569)	
Surgery past 6 months	Yes	6	7	1	NA	
	No	24	35	0.800 (0.239-2.677)		
Paternal occupation	Employee	11	16	1	1	
	Merchant	8	4	0.344 (0.083-1.429)	0.136 (0.14-1.324)	0.086
	Farmer	10	15	1.031 (0.340-3.126)	0.399 (0.027-5.815)	0.502
	Daily laborers	1	7	4.812 (0.517-14-822)	2.669 (0.149-9.061)	0.328
Maternal occupation	Housewife	19	25	1	NA	
	Employee	5	6	0.912 (0.242-3.442)		
	Merchant	6	11	1.393 (0.437 4.444)		
Paternal education	No education	8	18	1	1	
	Primary	10	12	0.533 (0.164-1.740)	0.294 (0.021-4.080)	0.362
	Secondary and above	12	12	0.444 (0.140-1.411)	0.309 (0.012-7.908)	0.478
Maternal education	No education	10	20	1	1	
	Primary	12	10	0.417 (0.134-1.292)	0.078 (0.003-2.384)	0.144
	Secondary and above	8	12	0.750 (0.232-2.424)	0.843 (0.020-14.961)	0.929
Family wealth index	Poor	4	10	1	1	
	Medium	23	19	0.330 (0.089-1.224)	0.029 (0.003-0.265)	0.002
	Rich	3	13	1.733 (0.314-9.573)	2.984 (0.144-12.696)	0.479

COR: crude odds ratio; AOR: adjusted odds ratio, ^∗^*P* value < 0.05, NA: not applicable, which is the variable did not meet criterion (*P* value < 0.25) to be included in multivariate analysis.

**Table 4:** Antibacterial resistance levels of *Enterobacteriaceae* isolated from children (<15 years) suspected of UTI in pediatric department at HUCSH from February 1 to July 30, 2018, Hawassa, Ethiopia.

Organism isolated	*N*	Antibiotics tested											
		AMC	AMP	CIP	GEN	STX	NIF	CTR	FOX	TET	NOR	CXT	MEM
*K. pneumonia*	20	19 (95)	19 (95)	7 (35)	17 (85)	17 (85)	7 (35)	17 (85)	11 (55)	17 (85)	8 (40)	15 (75)	11 (55)
*E. coli*	32	30 (93.8)	29 (90.6)	20 (62.5)	28 (87.5)	29 (90.6)	5 (15.6)	22 (68.8)	22 (68.8)	24 (75)	19 (59.4)	20 (62.5)	6 (18.8)
*K. ozaenae*	3	3 (100)	3 (100)	0 (0)	3 (100)	2 (66.7)	1 (33.3)	3 (100)	1 (33.3)	3 (100)	0 (0)	2 (66.7)	2 (66.7)
*K. oxytoca*	6	6 (100)	6 (100)	4 (66.7)	5 (83.3)	6 (100)	4 (66.7)	5 (83.3)	2 (33.3)	4 (66.7)	2 (33.3)	5 (83.3)	4 (66.7)
*E. cloacae*	2	2 (100)	2 (100)	1 (50)	2 (100)	2 (100)	0 (0)	1 (50)	1 (50)	1 (50)	0 (0)	1 (50)	0 (0)
*P. mirabilis*	2	2 (100)	2 (50)	0 (0)	2 (100)	2 (100)	0 (0)	0 (0)	2 (100)	1 (50)	1 (50)	0 (0)	0 (0)
*C. diversus*	3	3 (100)	3 (100)	1 (33.3)	2 (66.7)	2 (66.7)	0 (0)	2 (66.7)	2 (66.7)	2 (66.7)	2 (66.7)	2 (66.7)	2 (66.7)
*Providencia spp.*	4	4 (100)	4 (100)	1 (25)	3 (75)	2 (50)	2 (50)	4 (100)	2 (50)	2 (50)	1 (25)	3 (75)	4 (100)
Total	72	69 (95.8)	68 (94.4)	34 (47.2)	62 (86.1)	62 (86.1)	19 (26.4)	54 (75)	43 (59.7)	54 (75)	33 (45.8)	49 (68)	29 (40.3)

Abbreviations: AMC: amoxacillin/clavulanate; AMP: ampicillin; CIP: ciprofloxacin; GN: gentamicin; STX: trimethoprim-sulfamethoxazole; NIF: nitrofurantoin; CTR: ceftriaxone; FOX: cefoxitin; TET: tetracycline; NOR: norfloxacin; CXT: cefotaxime; MEM: meropenem.

**Table 5:** Multidrug resistance level of *Enterobacteriaceae* isolated from children (<15 years) suspected of UTI in pediatric department at HUCSH from February 1 to July 30, 2018, Hawassa, Ethiopia.

Level of antibiotics resistance (number (%))									Total MDR-E (>R3)	
Isolates (number)	R0	R1	R2	R3	R4	R5	R6	R7	
*E. coli* (32)	0 (0.0)	1 (3.1)	2 (6.3)	8 (25)	7 (21.9)	7 (21.9)	4 (12.5)	3 (9.4)	29 (90.6)
*K. pneumonea* (20)	0 (0.0)	1 (5.0)	2 (10.0)	8 (40.0)	4 (20.0)	3 (15.0)	1 (5.0)	1 (5.0)	17 (85.0)
*K. ozaenae* (3)	0 (0.0)	0 (0.0)	0 (0.0)	2 (66.7)	0 (0.0)	0 (0.0)	1 (33.3)	0 (0.0)	3 (100)
*K. oxytoca* (6)	0 (0.0)	1 (16.7)	1 (16.7)	1 (16.7)	0 (0.0)	2 (33.3)	0 (0.0)	1 (16.7)	4 (66.7)
*E. cloacae* (2)	0 (0.0)	0 (0.0)	0 (0.0)	1 (50.0)	1 (50.0)	0 (0.0)	0 (0.0)	0 (0.0)	2 (100)
*Citrobacter diversus* (3)	0 (0.0)	0 (0.0)	0 (0.0)	0 (0.0)	1 (33.3)	0 (0.0)	1 (33.3)	0 (0.0)	2 (66.7)
*P. mirabilis* (2)	0 (0.0)	0 (0.0)	0 (0.0)	0 (0.0)	1 (50.0)	1 (50.0)	0 (0.0)	0 (0.0)	2 (100)
*Providencia spp.* (4)	0 (0.0)	1 (25.0)	0 (0.0)	1 (25.00	0 (0.0)	2 (50.0)	0 (0.0)	0 (0.0)	3 (75.0)
Total (72)									62 (86.1)

**Table 6:** Antibacterial resistance levels of ESBL-producing and non-ESBL-producing *Enterobacteriaceae* among UTI-suspected children (<15 years) at HUCSH from February 1 to July 30, 2018, Hawassa, Ethiopia.

Drugs	ESBL producer (30) *n* (% of NS∗)	ESBL nonproducer (42) *n* (% of NS)	*P* value
Amoxicillin-clavulanic acid	29 (96.7)	40 (95.2)	0.63
Ampicillin	29 (96.7)	39 (92.9)	0.44
Ciprofloxacin	17 (56.7)	17 (40.5)	0.13
Gentamycin	29 (96.7)	33 (78.6)	0.028
Co-trimoxazole	29 (96.7)	33 (78.6)	0.028
Nitrofurantoin	12 (40)	7 (16.7)	0.026
Ceftriaxone	27 (90)	27 (64.3)	0.012
Cefoxitin	23 (76.7)	20 (47.6)	0.012
Tetracycline	26 (86.7)	28 (66.7)	0.047
Norfloxacin	20 (66.7)	13 (30.9)	0.003
Cefotaxime	29 (96.7)	20 (47.6)	<.0001
Meropenem	17 (56.7)	12 (28.6)	0.012

NS∗: nonsusceptible.

## Data Availability

Data can be provided by the principal investigator upon reasonable request.
